# Transorbital Craniocerebral Occult Penetrating Injury with Cerebral Abscess Complication

**DOI:** 10.1155/2012/742186

**Published:** 2012-10-14

**Authors:** Arif Abdulbaki, Faisal Al-Otaibi, Amal Almalki, Nasser Alohaly, Saleh Baeesa

**Affiliations:** ^1^Alfaisal University, Riyadh 11533, Saudi Arabia; ^2^Division of Neurological Surgery, Neurosciences Department, King Faisal Specialist Hospital and Research Center, Riyadh 11211, Saudi Arabia; ^3^Division of Ophthalmology, Department of Surgery, King Faisal Specialist Hospital and Research Center, Riyadh 11211, Saudi Arabia; ^4^Division of Neurological Surgery, Faculty of Medicine, King Abdulaziz University, Jeddah 21589, Saudi Arabia

## Abstract

Transorbital intracranial penetrating injury is an uncommon mechanism of head injury. These injuries can be occult during the initial clinical presentation. Certain patients develop an intracranial cerebral infection. Herein, we report a 5-year-old child with an occult transorbital intracranial penetrating injury caused by a pen. A retained pen tip was found at the superior orbital roof and was not noticed at initial presentation. This was complicated by a right frontal lobe cerebral abscess. This paper emphasizes the importance of orbitocranial imaging in any penetrating orbital injury. A review of the literature on intracranial infection locations in relation to the route and mechanism of injury is included to complement this report.

## 1. Introduction

Transorbital penetrating craniocerebral injuries account for 24% of penetrating head injuries in adults and around 45% in children [1, 2]. This unusual injury is associated with intracranial complications, such as brain abscess, meningitis, cerebrospinal fluid leakage, hemorrhage, neurological deficit, and mortality [3]. Cerebral infection is found to be the most common cause of mortality [4]. There are thin bones in the skull, including the orbital walls, which are known to be access routes for slow-penetrating foreign bodies to enter the craniocerebral compartment. Different types of foreign bodies with variable transorbital routes of entry into the cranial cavity have been discussed in the literature [5]. Occult penetrating injuries happen when there is a small laceration at the orbital soft tissue without any other clinical findings. This lack of findings leads to delayed diagnosis of the foreign body injury, and, moreover, subsequent complications, such as a brain abscess, can occur. Here, we report a child who sustained a penetrating transorbital craniocerebral injury by a pen. Delayed diagnosis led to frontal abscess formation. The routes of injury and infection locations are discussed based on a review of the available literature.

## 2. Case Report

**Figure 1:**
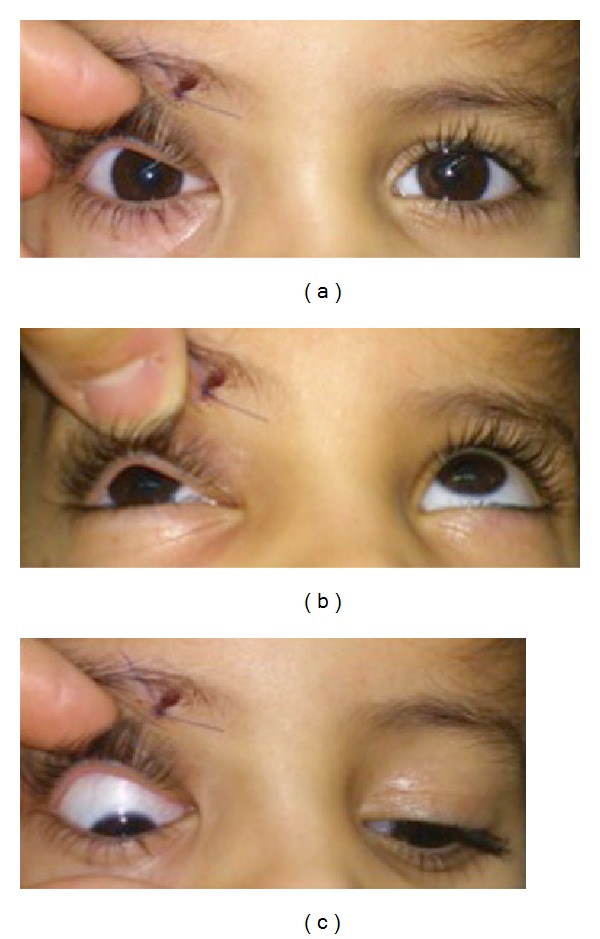
Patient gaze assessment photographs demonstrating the sutured site of foreign body penetration, slight downward right eye deviation on primary gaze (a), limitation of upward gaze (b), and exaggerated downward gaze (c).

**Figure 2:**
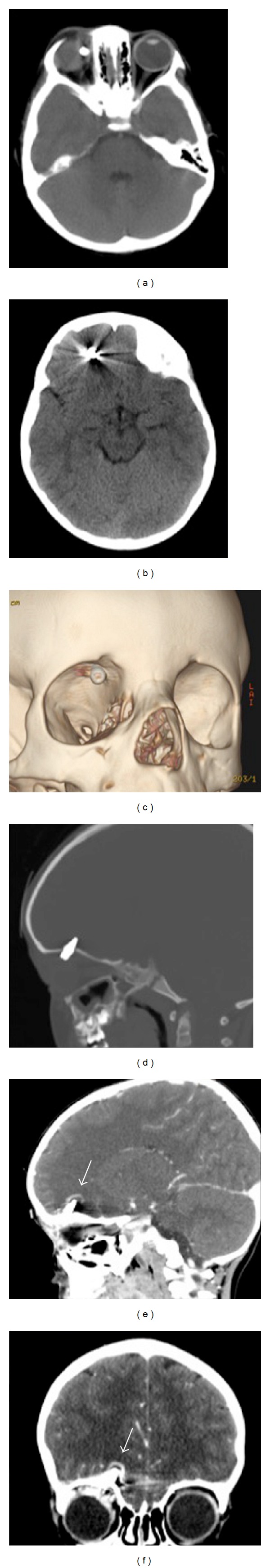
Computed tomography (CT) scans of the brain and orbits depicting the foreign body within the orbit and right frontal lobe (a) and (b). The medial orbital roof foreign body penetration site is shown in 3D CT (c). The foreign body shape (pen's tip) and the surrounding rim enhancement indicating the presence of abscess is shown in figures (d), (e), and (f).

A 5-year-old female fell down on a pen while playing. The tip of the pen became lodged inside the right orbitocranial region. Her parents did not witness this injury initially, and there was no history of any neurological symptoms. At first, she was treated in a local clinic with simple suturing of the laceration at the upper eyelid (Figure 1). Four days later, she presented to a local hospital with orbital swelling without fever where she was found to have a retained orbitocranial foreign metallic body. She was started on antibiotics and subsequently referred to our hospital for further management. Upon arrival, she was complaining of pain from the right eye with no associated neurological symptomatology. On clinical examination, she was afebrile and all other vital signs were stable. Sutured laceration was noted at the superior medial upper eyelid. Ophthalmological examination revealed restriction of the upward gaze due to muscle entrapment; the globe was slightly pushed downward due to the swelling and muscle entrapment (Figure 1). No cerebrospinal fluid (CSF) leakage was noted. She had a Glasgow coma score (GCS) of 15 with no neurological dysfunction. Computed tomography (CT) scans of the brain and orbits showed a 20 mm × 15 mm metallic foreign body attached in the medial portion of the right orbital roof, representing the pen tip (Figure 2). The foreign body's distal end was penetrating the right frontal lobe inferior surface, whereas the proximal end was situated at the upper orbital cavity. A rim-like enhancement was seen at the frontal lobe around the foreign body tip suggestive of an abscess formation surrounded by cerebral edema (Figure 2). Her laboratory investigations did not reveal any leukocytosis in the blood, and the result of the blood culture was negative. The patient was kept on an antibiotic regiment of vancomycin, ceftazidime, and metronidazole.

**Figure 3:**
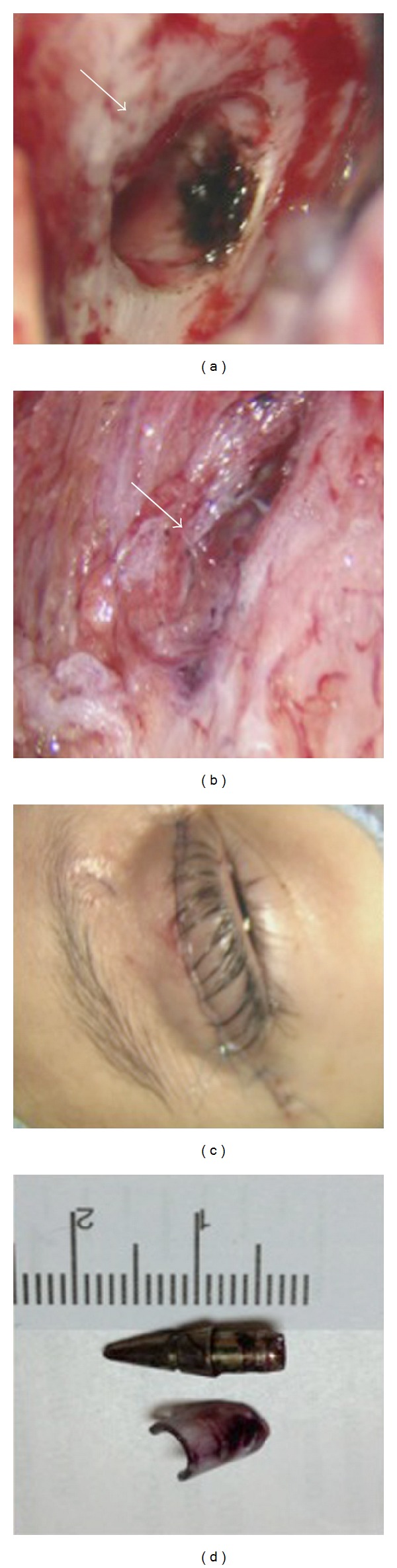
Intraoperative photographs demonstrating the following. (a) The site of orbital roof defect caused by the penetrating foreign body (arrow). (b) The penetrating wound at the orbital muscles and the pen ink (arrow). (c) The surgical approach (transeyelid) site after closure. (d) The penetrating pen's tip and the piece of glass from the pen shaft.

After one day of being admitted to our hospital, she underwent foreign body removal and debridement of the brain abscess. Transcutaneous upper eyelid surgery was carried out by a team that included both oculoplastic and neurological surgeons. Upper eyelid skin crease was opened exploring the superior subperiosteal space, exposing the proximal end of the foreign body that comprised the pen's metallic tip and pieces of glass from the pen's shaft (Figure 3). The pen tip was attached to the superior orbital roof, and after removal, CSF leakage and minimal purulent discharge were noted. The dura was opened, and the pen tip penetrated the frontal lobe inferior surface. Ink from the pen was noted on the brain and orbital muscles. Debridement and irrigation were performed at the tract of the penetrating wound. A small piece of surgical patch was placed between the bone and the dural opening to prevent CSF leakage into the orbit. Finally, the wound was closed and dressing was applied (Figure 3).

**Figure 4:**
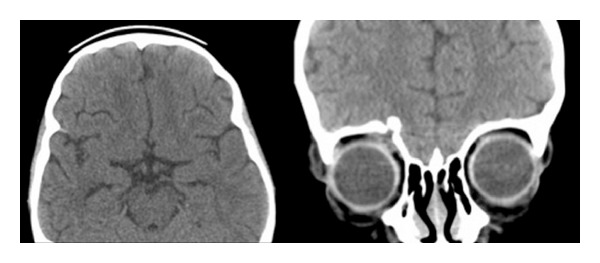
Computed tomography (CT) scans of the cranium demonstrating the resolution of frontal edema and abscess 8 weeks after treatment.

Postoperatively, she had transient periorbital swelling. The culture from the brain abscess was negative. This was likely due to the antibiotic treatment initiated prior to this patient's transfer to our hospital. Subsequently, she had full extraocular movement with improvement of the upper-gaze limitation, and the wound healed well. She was last seen 2 months after the surgery, and she was having mild right-eye ptosis. Her CT scan followup showed resolution of the brain abscess and edema after a course of 3 weeks of antibiotics (Figure 4). 

## 3. Discussion

Penetrating cranial injury is an uncommon type of head injury. It constitutes 0.4% of all head injuries [16]. There are different routes for penetrating foreign bodies to enter the cranial cavity through the orbit. The most common route of entry is the superior orbital roof [5, 17]. Moreover, there are certain areas within the orbit that provide direct access to the intracranium, such as the superior orbital fissure, inferior orbital fissure, and optic canal. Turbin and colleagues analyzed the pattern of transorbital intracranial injury and divided the orbital surface into different zones [5]. These different routes of transorbital injury are associated with different locations of craniocerebral injury. Balasubramanian and colleagues classified transorbital penetrating injury based on the orbital bone's anatomy and the associated injury [18]. This analysis of injury patterns could help in tailoring management and surgical approach, as well as in anticipating the potential type and site of intracranial complications related to foreign body penetration. 

In certain cases of orbital penetrating injury, the foreign body might be missed and go undiagnosed. In such cases, eyelid laceration is sutured without further investigation [3]. This delayed diagnosis of the penetrating foreign body is usually associated with more complications, such as orbital cellulitis, cerebral abscess, meningitis, and even delayed neurological deterioration related to slowly expanding intracranial hematoma [5, 19]. Mortality has been reported to be high in old literatures due to infections and lack of optimal antimicrobial therapy [4]. Rarely, delayed diagnosis of a retained foreign body can be made after many years of the injury [8]. Therefore, early recognition of foreign bodies by appropriate radiological images will indeed prevent life-threatening complications. Based on several case reports and small case series, the most common site of cerebral abscess formation occurs around the distal tip of the foreign body [5]. These intracranial abscess formations can be distal from the orbit and located as far as the cerebellum and prepontine areas [10, 15].  Table 1 summarizes studies of transorbital penetrating injuries associated with cerebral infections.

Surgical approaches are tailored according to the intracranial- and intraorbital-associated injuries and types of complications. In our case, we elected to approach the foreign body through the upper eyelid (oculoplastic approach) and debride the tract of the penetrating wound from the eyelid to the intracranial distal part. Furthermore, the base of the foreign body was attached to the superior orbital roof with the base in the orbit and tip within the frontal lobe. The small cerebral abscess was drained using this approach. The surgical culture was negative in our patient. This is likely due to the use of antibiotics prior to transfer to our hospital. In the literature, the commonly reported pathogens for abscess formation or meningitis are *Streptococcus* and *Staphylococcus* [5]. In general, the overall outcome of this type of injury is dependent on the degree and types of damage caused by the foreign body penetration. However, early recognition would prevent complications that can be life threatening.

## 4. Conclusion

Occult transorbital intracranial penetrating injury is an unusual form of trauma that is associated with significant morbidity. High suspension index of the presence of retained foreign body is mandatory in emergency medicine and appropriate radiological images should be considered.

## Figures and Tables

**Table 1:** Summary of selected studies for transorbital craniocerebral penetrating injuries complicated by cerebral infection.

Author (year)	Age (year)	Sex	Route of entry into cranial cavity	Foreign body type	Cerebral abscess location and infection type	Surgical approach	Outcome findings
Seider et al. (2006) [6]	1	Male	orbital roof	Pencil tip (made of graphite)	Frontal lobe abscess	Frontal burr hole and orbitotomy	Right upper eyelid ptosis
Maruya et al. (2002) [7]	56	Female	Lateral orbital wall	Bamboo fragments	Left temporal lobe abscess	Left frontotemporal craniotomy and orbito-zygomatic osteotomy	Slight left-eye lateral gaze limitation
Aulino et al. (2005) [8]	35	Male	Left middle cranial fossa	Fiberglass	Anterior left temporal lobe	Left pterional craniotomy	No neurologic deficit
Santoreneos et al. (1997) [9]	12	Male	Superior orbital fissure	Wooden foreign body (tree branch)	Medial aspect of the right temporal lobe	Right fronto-temporal craniotomy	Right eye loss of vision due to trauma, ptosis, and seizure
Matsuyama et al. (2001) [10]	1	Male	Superior orbital fissure	Chopstick	Prepontine area	Right frontolateral craniotomy	No neurological deficits
di Roio et al.(2000) [11]	6	Male	Orbital roof	Chopstick	Left frontal lobe	Abscess aspiration	No documented abnormalities
Rahman et al. (1997) [12]	30	Male	Superior orbital fissure	Nail	Meningitis	Extradural pterional craniotomy	Right-eye blindness
Potapov et al. (1996) [13]	26	Male	Medial orbital wall	Wooden foreign body	Right temporal lobe	Fronto-temporal craniotomy	Loss of visual function; right-sided ptosis
Specht et al. (1992) [14]	9	Male	Optic canal	Wooden golf tee	Meningitis	Fronto-temporal craniotomy	Some weakness of the face and extremities on the left side
Amano and Kamano (1982) [15]	7	Male	Superior orbital fissure	Bamboo stem	Meningitis and right cerebellar abscess	No surgery only antibiotics	Gradual reduction the cerebellar abscess size
Present case	5	Female	Orbital roof	Pen	Frontal lobe abscess	Transcutaneous upper eyelid approach	Mild right-eye ptosis
